# The Effect of Statin Therapy on the Overall Survival of Patients with Hepatocellular Carcinoma: A Single-Center Experience

**DOI:** 10.3390/cancers18132138

**Published:** 2026-07-02

**Authors:** Konstantinos Papantoniou, Vasileios Lekakis, Efthymios P. Tsounis, Evangelia Bourdalou, Nikitas Kimiskidis, Georgios Geramoutsos, Ploutarchos Pastras, Ioanna Aggeletopoulou, Odyssefs Ampazis, Georgia Diamantopoulou, Fotis Chrysanthakopoulos, Angelos Koutras, Tryfon Spyridonidis, Konstantinos Katsanos, Konstantinos Thomopoulos, Christos Triantos

**Affiliations:** 1 Division of Gastroenterology, Department of Internal Medicine, University of Patras, 26504 Patras, Greece; med6162@ac.upatras.gr (K.P.); up1081417@upatras.gr (E.P.T.); ebourdalou@gmail.com (E.B.); nkimiskidis93@gmail.com (N.K.); giorgosgeramoutsos@gmail.com (G.G.); med6438@ac.upatras.gr (P.P.); iaggel@upatras.gr (I.A.); odysampazis@gmail.com (O.A.); geodiamant@hotmail.com (G.D.); thomkons@upatras.gr (K.T.); 2 First Academic Department of Gastroenterology, Medical School of National and Kapodistrian University of Athens, General Hospital of Athens “Laiko”, 11527 Athens, Greece; vlekakis@med.uoa.gr; 3 Division of Oncology, Department of Medicine, University Hospital of Patras, 26504 Patras, Greece; fotis19257@gmail.com (F.C.); angkoutr@upatras.gr (A.K.); 4 Nuclear Medicine Department, University Hospital of Patras, Rio, 26504 Patras, Greece; tspyr@med.upatras.gr; 5 Department of Interventional Radiology, University Hospital of Patras, Rio, 26504 Patras, Greece; katsanos@med.upatras.gr

**Keywords:** statins, hepatocellular carcinoma, tumor stage, comorbidities, mortality

## Abstract

**Background**: Statins have pleiotropic anti-inflammatory, antifibrotic, and potential antineoplastic effects. Although several studies have linked statin exposure to lower hepatocellular carcinoma (HCC) incidence, their effect on survival after HCC diagnosis remains uncertain. We evaluated whether statin therapy before HCC diagnosis was associated with overall survival in a real-world cohort. **Methods**: We performed a retrospective single-center cohort study of consecutive patients with HCC managed at a tertiary referral center between January 2000 and January 2025. Demographic, clinical, laboratory, tumor-related, and treatment variables were collected at diagnosis and during follow-up. Patients were classified according to statin use before HCC diagnosis. Overall survival (OS) was assessed using Kaplan–Meier analysis and Cox proportional hazards regression. **Results**: Overall, 190 patients were included; 172 (90.5%) were male, 136 (71.6%) had cirrhosis, and 42 (22.1%) received statins. Statin users more frequently had diabetes mellitus, elevated body mass index and arterial hypertension, but baseline liver function, alpha-fetoprotein levels, tumor burden, and treatment allocation were broadly comparable between groups. Among 159 patients with available survival data, statin users had longer OS than non-users (mean 82.5 vs. 41.7 months; median 57 vs. 31 months; log-rank *p* < 0.001). In univariate Cox analysis, statin therapy was associated with reduced mortality risk (HR 0.45, 95% CI 0.28–0.70, *p* < 0.001). This association remained significant across multivariable models adjusting for baseline liver function, tumor stage, vascular invasion, and diagnostic era. **Conclusions**: Pre-diagnostic statin therapy was independently associated with improved OS in patients with HCC. These hypothesis-generating findings warrant prospective multicenter validation and careful assessment of confounding by indication.

## Simple Summary

Statins are widely used medications for the prevention of cardiovascular disease. Several studies suggest that they may also have anti-inflammatory and anticancer properties. In this retrospective single-center study, we evaluated whether statin therapy before hepatocellular carcinoma (HCC) diagnosis was associated with survival outcomes. Patients receiving statins before HCC diagnosis had significantly longer overall survival compared with non-users, even after adjustment for factors that are commonly associated with HCC prognosis, such as liver function, tumor burden, and treatment-related factors. These findings suggest that statins may have a beneficial role in patients with HCC and support the need for further prospective studies.

## 1. Introduction

Hepatocellular carcinoma (HCC) is the most common primary liver malignancy and remains a major cause of cancer-related mortality worldwide. Most cases arise in the setting of chronic liver disease and cirrhosis, particularly in patients with chronic hepatitis B or C virus infection, alcohol-related liver disease, or metabolic dysfunction-associated steatotic liver disease (MASLD) [1,2]. Significant advances have been made in surveillance and therapeutic options, which include surgical resection, transplantation, regional interventional therapies, and newer immunotherapeutic agents [3]. However, the prognosis of HCC remains poor, with studies indicating 5 year survival rarely exceeds 50% [4].

Moreover, recent epidemiological data indicate that the incidence of HCC-related mortality will continue to increase in the next decades, while a shift in etiology will also be observed. Although viral-hepatitis-related deaths used to be much more common, alcohol-related liver disease (ALD) and MASLD are emerging as growing etiologies, with ALD expected to become the leading cause of HCC-related deaths by 2026, followed by MASLD in 2032 [5].

Statins are 3-hydroxy-3-methylglutaryl coenzyme A reductase inhibitors that are widely used for dyslipidemia and cardiovascular risk reduction. Beyond lipid lowering, statins exert pleiotropic immunomodulatory, anti-inflammatory, antioxidant, and antifibrotic effects [6]. These effects have increased the interest in their potential benefit in chronic liver disease. In MASLD, these properties have raised interest in a potential role for statins in reducing fibrosis progression, portal hypertension, hepatic decompensation, and liver-related mortality [7,8,9].

Preclinical and clinical data also suggest that statins may have anticancer activity through effects on the mevalonate pathway, oncogenic signaling, angiogenesis, apoptosis, and the tumor microenvironment [10]. Several observational studies and meta-analyses have reported lower HCC incidence among statin users [11], and recent data suggest that statin use after curative HCC treatment may be associated with improved survival [12]. However, evidence regarding the association between statin use and survival outcomes after HCC diagnosis remains heterogeneous, particularly in real-world cohorts with detailed information on liver function, tumor characteristics, and treatment allocation.

The aim of the present study was to investigate whether pre-diagnostic statin therapy was associated with overall survival among patients with HCC managed at a tertiary referral center. We further explored whether this association persisted after adjustment for baseline liver function, tumor burden, and the era of HCC diagnosis.

## 2. Materials and Methods

### 2.1. Study Design and Participants

This was a non-interventional, retrospective, single-center cohort study including consecutive patients with HCC of any etiology who were managed at the Division of Gastroenterology, Department of Internal Medicine, University Hospital of Patras, Greece, between January 2000 and January 2025. Because the study period extended over many years, the diagnosis of HCC was established according to internationally accepted guidelines applicable at the time of diagnosis. In most cases, HCC was diagnosed non-invasively based on characteristic findings on contrast-enhanced ultrasound (CEUS), multiphasic computed tomography (CT), or magnetic resonance imaging (MRI), including arterial phase hyperenhancement and washout on portal venous and/or delayed phase imaging where applicable. Serum alpha-fetoprotein (AFP) levels were also evaluated as part of the diagnostic workup. Histological confirmation was obtained in cases with inconclusive imaging findings or when otherwise clinically indicated. HCC management was based on the evidence-based recommendations and institutional multidisciplinary practice applicable during each treatment period.

This study conforms to the ethical principles outlined in the Declaration of Helsinki for medical research involving human subjects [13]. Anonymized data from routine clinical care were used in the analysis. According to institutional policy, formal informed consent was not required.

### 2.2. Patient Data and Variables

Demographic, clinical, laboratory, tumor-related, and treatment data were retrospectively collected at the time of HCC diagnosis and during follow-up. Variables included age, sex, body mass index (BMI), smoking and alcohol history, diabetes mellitus, arterial hypertension, dyslipidemia, alpha-fetoprotein (AFP), cirrhosis, hepatic decompensation, ascites, variceal bleeding, Child-Pugh score, Model for End-stage Liver Disease Sodium (MELD-Na) score, tumor multiplicity, portal vein thrombosis (PVT), extrahepatic metastasis, Milan criteria status, and Barcelona Clinic Liver Cancer (BCLC) classification where available.

HCC-directed treatments were recorded, including hepatectomy, transarterial chemoembolization (TACE), radiofrequency ablation (RFA), sorafenib, atezolizumab/bevacizumab, lenvatinib, regorafenib, cabozantinib, and nivolumab. Statin exposure was defined a priori as documented statin therapy before the date of HCC diagnosis, which represented the baseline time point and time zero for survival analysis. Patients were classified as statin users or non-users according to this pre-diagnostic exposure status. No post-diagnostic survival time was required for classification as a statin user, and statin use was therefore analyzed as a baseline exposure variable.

### 2.3. Main Outcome

The primary outcome was overall survival (OS), defined as the interval from HCC diagnosis to death from any cause or the last available follow-up. Survival status was determined from medical records and available follow-up information. Patients for whom no information regarding vital status was available after their last evaluation at our center were considered lost to follow-up and were excluded from the survival analyses.

### 2.4. Statistical Analysis

Statistical analyses were performed using IBM SPSS Statistics version 29.0 (IBM Corp., Armonk, NY, USA). Continuous variables were assessed for distributional characteristics and are presented as mean +/− standard deviation (SD) or median with interquartile range (IQR), as appropriate. Categorical variables are summarized as frequencies and percentages. Comparisons between statin users and non-users were performed using the independent-samples t-test or Mann–Whitney U test for continuous variables and the chi-square test or Fisher exact test for categorical variables, as appropriate. Given the retrospective design and variable-specific missingness, available-case analyses were used for baseline comparisons.

OS was estimated using the Kaplan–Meier method, and survival distributions were compared with the log-rank test. Univariable Cox proportional hazards regression was used to evaluate candidate predictors of mortality. Multivariable Cox regression models were then constructed using clinically relevant variables and variables associated with survival in univariable analysis. Results are reported as hazard ratios (HRs) with 95% confidence intervals (CIs). A two-sided *p*-value < 0.05 was considered statistically significant.

To minimize the risk of immortal time bias and other time-related biases, multivariable Cox regression models were restricted to variables available at the time of HCC diagnosis. HCC-directed treatments administered after diagnosis were not included because treatment allocation might depend on survival time, disease evolution, and changes in clinical practice during follow-up. Moreover, given the long study period and the substantial evolution of HCC management over time, diagnostic era was included as an additional baseline covariate in sensitivity analyses and categorized according to the calendar period of HCC diagnosis as follows: 1 = 2000–2010, 2 = 2011–2017, and 3 = 2018–2025. This variable was defined at baseline and was added to multivariable Cox regression models to account for potential temporal confounding. Two baseline-adjusted models were constructed: Model 1 included pre-diagnostic statin use, BCLC stage at baseline, MELD-Na score at baseline and diagnostic era; Model 2 included pre-diagnostic statin use, BCLC stage at baseline, Child-Pugh score at baseline, PVT at baseline, and diagnostic era.

## 3. Results

### 3.1. Patient Characteristics at Baseline

A total of 190 patients with HCC were included. The cohort was predominantly male (172/190, 90.5%), with a median age at HCC diagnosis of 66 years (IQR 58.5–72). Cirrhosis was present in 136 patients (71.6%), and hepatic decompensation at baseline was documented in 47 patients (24.7%). Pre-diagnosis statin therapy was documented in 42 patients (22.1%). Dyslipidemia was recorded in 34 patients, while an additional 8 patients were receiving statins for secondary cardiovascular prevention despite the absence of a documented dyslipidemia diagnosis. All these patients continued to receive statin therapy during follow-up. Baseline characteristics, tumor features, and subsequent treatments are summarized in Table 1.

### 3.2. Baseline Patient Characteristics According to Statin Use

Baseline characteristics were compared according to statin exposure (Table 2). Statin users had a higher prevalence of diabetes mellitus (47.6% vs. 25.0%, *p* = 0.005), BMI (29.1 vs. 25.8 kg/m^2^, *p* = 0.006) and arterial hypertension (64.3% vs. 42.4%, *p* = 0.012), consistent with the cardiovascular indications for statin therapy. There were no statistically significant differences in etiology of liver disease, cirrhosis, hepatic decompensation, ascites, variceal bleeding, PVT, extrahepatic metastasis, Child-Pugh score, MELD-Na score, AFP levels, or Milan criteria status. Statin users more frequently had single tumors, while treatment allocation, including hepatectomy, TACE, RFA, and systemic therapies, did not differ significantly between groups. These findings indicate comparable baseline liver function, tumor burden, and treatment choices between the two groups.

### 3.3. Survival Analysis

**Figure 1:**
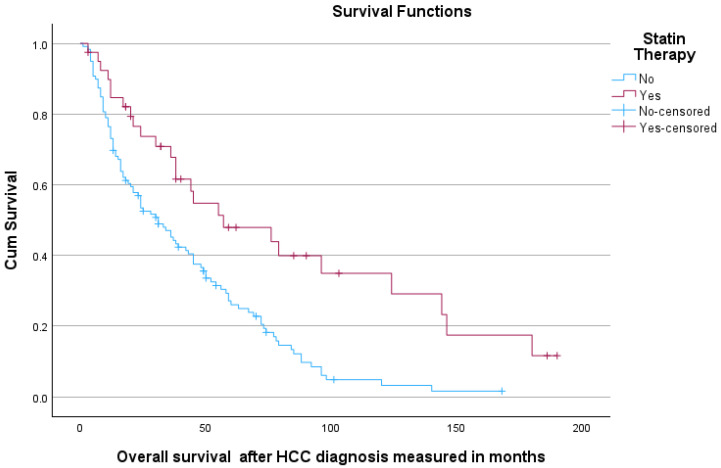
Kaplan–Meier survival curves comparing overall survival between statin users and non-users. Patients receiving statins before HCC diagnosis demonstrated significantly longer survival (log-rank *p* < 0.001).

Of the 190 patients included in the study, survival data were available for 159 patients. The remaining 31 patients were excluded from survival analyses because they were lost to follow-up and no information regarding their vital status was available after their last evaluation at our center. In the overall cohort with available survival information, mean OS was 42.1 months (95% CI 36.1–48.2), and median OS was 31 months. Kaplan–Meier analysis demonstrated significantly longer survival among statin users compared with non-users (Figure 1). Mean OS was 82.5 months (95% CI 59.7–105.4) in statin users and 41.7 months (95% CI 34.8–48.6) in non-users, while median OS was 57 months and 31 months, respectively. The difference in survival distributions was statistically significant (log-rank chi-square = 12.842, *p* < 0.001).

In univariable Cox regression analysis, statin therapy was associated with a significantly lower risk of mortality (HR 0.45, 95% CI 0.28–0.70, *p* < 0.001). Adverse prognostic factors included hepatic decompensation, variceal bleeding, ascites, PVT, and extrahepatic metastasis, whereas hepatectomy and RFA were associated with improved survival (Table 3).

### 3.4. Multivariate Cox Regression

Statin therapy remained independently associated with improved survival in multivariable Cox regression models. In a model including statin therapy, BCLC classification at baseline, MELD-Na score at baseline, and the era of HCC diagnosis, statin use was associated with reduced mortality risk (HR 0.44, 95% CI 0.20–0.99, *p* = 0.047), while higher MELD-Na score independently predicted mortality (HR 1.16, 95% CI 1.06–1.28, *p* = 0.002) (Table 4).

In a broader model that additionally included BCLC classification at diagnosis, Child-Pugh score, PVT, and the era of HCC diagnosis, statin therapy remained strongly associated with improved survival (HR 0.36, 95% CI 0.19–0.68, *p* = 0.002). Child-Pugh score and PVT at baseline independently predicted worse survival (Table 5).

## 4. Discussion

In the present study, we demonstrated that statin therapy was independently associated with significantly improved overall survival in patients with HCC. Importantly, this association remained significant across multiple multivariate Cox regression models, even after adjustment for established liver function markers, tumor burden, and therapeutic era. The observed survival benefit was not only statistically significant but also clinically meaningful, with statin users exhibiting markedly prolonged median and mean overall survival compared with non-users. These findings support the growing hypothesis that statins may exert clinically relevant antineoplastic and hepatoprotective effects in patients with HCC [14].

One of the major strengths of the present study lies in the detailed characterization of a real-world HCC population followed over a prolonged period during which substantial changes in hepatology and oncology practice took place. Our cohort included patients managed across different therapeutic eras, ranging from earlier periods dominated by surgical and locoregional approaches to more recent years characterized by the introduction of systemic targeted therapies and immunotherapy [15]. Despite this heterogeneity in treatment evolution and clinical management, pre-diagnostic statin use remained independently associated with improved survival across the adjusted analyses. This finding is particularly important, as it suggests that the observed association between statin use and improved survival might extend beyond a specific therapeutic setting and may remain relevant even in the context of modern multidisciplinary HCC management. Furthermore, our analysis was based on a well-characterized cohort with detailed clinical, laboratory, radiological, and therapeutic data, allowing for a more comprehensive adjustment for potential confounders.

The potential mechanisms underlying the beneficial effects of statins in HCC are likely multifactorial. Statins inhibit hydroxymethylglutaryl-coenzyme A (HMG-CoA) reductase, thereby suppressing the mevalonate pathway, which plays a central role not only in cholesterol synthesis but also in the de-activation of molecules involved in the inflammatory process, such as the Nuclear factor kappa-light-chain-enhancer of activated B cells (NF-κB) pathway and the NOD-, LRR- and pyrin domain-containing protein 3 (NLRP3) inflammasome [16]. Experimental studies have demonstrated that statins may inhibit tumor cell proliferation, induce apoptosis, impair angiogenesis, and reduce metastatic potential in many types of malignancies through the disruption of several signaling pathways [17,18,19]. In addition, statins appear to modulate several molecular pathways implicated in hepatocarcinogenesis, including Ras/Rho signaling, MYC activation, and pathways associated with cellular proliferation and oxidative stress [10,20,21]. Further studies suggest that statins could limit the incidence and evolution of several malignancies, even in advanced stages [22].

Beyond their direct antitumor effects, statins may also exert beneficial effects through modulation of the underlying chronic liver disease. Several studies have suggested that statins may influence the tumor microenvironment and inflammatory cascades involved in chronic liver injury, therefore reducing fibrosis progression and cirrhosis development [23]. Increasing evidence supports a favorable role of statins in MASLD, where statin use has been associated with reduced fibrosis progression, lower portal hypertension, and potentially reduced HCC risk [24]. Similarly, beneficial associations have been reported in chronic viral hepatitis, particularly hepatitis B and hepatitis C infection, where statins have been linked to lower rates of hepatic decompensation, fibrosis progression, and HCC development [25,26]. Emerging evidence also suggests potential anti-inflammatory and antifibrotic effects in cholestatic liver diseases such as PBC [27]. Taken together, these findings support the concept that statins may exert broad hepatoprotective effects extending beyond lipid lowering alone.

Our findings are in agreement with several previous observational studies and meta-analyses suggesting improved outcomes among HCC patients receiving statins. Large population-based studies have demonstrated associations between statin exposure and reduced HCC incidence [28], while more recent analyses have suggested improved survival among patients with established HCC and the addition of statins to different treatment options, including hepatectomy [12] and RFA [29]. However, available evidence remains heterogeneous, with differences in study design, patient populations, etiological backgrounds, statin type and dose, and adjustment strategies. Moreover, relatively few studies have specifically evaluated the long-term impact of statins on overall survival in carefully phenotyped HCC cohorts with detailed adjustment for liver function and therapeutic interventions. In this context, our study contributes additional real-world evidence supporting a possible association between statin therapy and improved survival in patients with HCC.

An important observation in our cohort was that baseline liver function and tumor severity were generally comparable between statin users and non-users. Child–Pugh score, MELD-Na score, and AFP levels did not significantly differ between the two groups, reducing the likelihood that the observed survival benefit was exclusively driven by major baseline differences in disease burden. Although patients receiving statins more frequently exhibited metabolic comorbidities such as diabetes mellitus, elevated BMI and arterial hypertension, these characteristics would generally be expected to be connected with a worse rather than better prognosis [30]. Therefore, the favorable survival outcomes observed among statin users are unlikely to be fully explained by differences in baseline disease severity and may support a beneficial association between statin exposure and survival.

Some limitations of our study should be acknowledged. First, the study was conducted at a single tertiary center, which may limit external validity. An additional limitation relates to the potential for time-related bias inherent to retrospective survival analyses. Although statin exposure was defined before HCC diagnosis and assigned at baseline, thereby minimizing the risk of immortal time bias related to post-diagnostic statin initiation, detailed longitudinal information on statin dose, adherence, discontinuation, and post-diagnostic exposure changes was not consistently available. Therefore, a formal time-dependent statin exposure analysis could not be performed. Moreover, HCC-directed treatments delivered after diagnosis may be influenced by survival time, disease evolution, and changes in therapeutic practice over the long study period. For this reason, post-diagnostic treatment variables were excluded from the revised multivariable Cox models, which were restricted to baseline liver function and tumor-related prognostic factors. These limitations preclude causal inference, and the observed association should be interpreted as hypothesis-generating. Finally, the study period extended from 2000 to 2025, during which HCC diagnosis, staging, surveillance, locoregional therapies, systemic therapy, immunotherapy, and supportive care evolved substantially. Although we attempted to address this issue by including diagnostic era as a baseline covariate in sensitivity analyses, residual temporal confounding cannot be fully excluded.

Despite these limitations, the study has several strengths. It includes a real-world HCC population with long follow-up and detailed clinical, tumor-related, and treatment information. The survival signal was robust across multiple Cox regression models and remained evident after adjustment for recognized prognostic factors. These findings support further investigation of statins in prospective multicenter cohorts and, ideally, in carefully designed interventional or pragmatic trials focusing on statin type, dose, duration, safety, and patient selection.

## 5. Conclusions

In this single-center retrospective cohort, statin therapy before HCC diagnosis was independently associated with improved overall survival. The association persisted after adjustment for liver function, tumor stage, vascular invasion, and diagnostic era. While these findings support further investigation of statins as a potential adjunctive strategy in patients with HCC, prospective multicenter studies are required to validate the results, clarify causality, and determine whether specific statin strategies can improve outcomes in patients with HCC.

## Figures and Tables

**Table 1:** Patient characteristics at baseline and subsequent treatment.

Variable	Value
Demographics and statin use	
Male sex, *n* (%)	172 (90.5)
Age at HCC diagnosis, median (IQR)	66.0 (58.5–72.0)
Active smoking, *n* (%)	65 (34.2)
Active alcohol abuse, *n* (%)	35 (18.4)
Prior alcohol abuse, *n* (%)	43 (22.6)
BMI (kg/m^2^), median (IQR)	26.1 (23.9–29.1)
Statin use, *n* (%)	42 (22.1)
Comorbidities	
Diabetes mellitus, *n* (%)	57 (30.0)
Arterial hypertension, *n* (%)	88 (46.3)
Dyslipidemia, *n* (%)	34 (17.9)
Liver Disease	
Cirrhosis, *n* (%)	136 (71.6)
Hepatic decompensation, *n* (%)	47 (24.7)
Ascites, *n* (%)	42 (22.1)
Variceal bleeding, *n* (%)	15 (7.9)
Tumor Characteristics	
Single tumor, *n* (%)	86 (45.3)
PVT, *n* (%)	23 (12.1)
Extrahepatic metastases, *n* (%)	8 (4.2)
Within Milan criteria, *n* (%)	71 (37.4)
Tumor Burden	
Child–Pugh score, mean ± SD	5.82 ± 1.21
MELD-Na score, mean ± SD	10.68 ± 3.67
AFP at baseline (ng/mL), median (IQR)	280.0 (74.5–2225.0)
Treatment Modalities	
Hepatectomy, *n* (%)	35 (18.4)
TACE, *n* (%)	74 (38.9)
RFA, *n* (%)	58 (30.5)
Sorafenib, *n* (%)	47 (24.7)
Atezolizumab/Bevacizumab, *n* (%)	13 (6.8)
Lenvatinib, *n* (%)	7 (3.9)
Regorafenib, *n* (%)	12 (6.3)
Cabozantinib, *n* (%)	11 (5.8)
Nivolumab, *n* (%)	6 (3.2)

Abbreviations: HCC, hepatocellular carcinoma; SD, standard deviation; IQR, interquartile range; PVT, portal vein thrombosis; AFP, alpha-fetoprotein; MELD-Na, Model for End-stage Liver Disease Sodium; TACE, transarterial chemoembolization; RFA, radiofrequency ablation.

**Table 2:** Baseline Characteristics and Treatment Allocation According to Statin Use.

Variable	Statin Users (*n* = 42)	Non-Users (*n* = 148)	*p*-Value
Demographics			
Male sex, *n* (%)	40 (95.2)	132 (89.2)	0.233
Age at HCC diagnosis, median (IQR)	68.5 (59.5–74.5)	66.0 (57.0–72.0)	0.298
BMI (kg/m^2^), median (IQR)	29.1 (24.7–32.5)	25.8 (23.7–27.4)	0.006
Active smoking, *n* (%)	17 (40.5)	48 (32.4)	0.837
Comorbidities			
Diabetes mellitus, *n* (%)	20 (47.6)	37 (25.0)	0.005
Arterial hypertension, *n* (%)	27 (64.3)	61 (42.4)	0.012
Liver Disease			0.770
HBV, *n* (%)	17 (40.5)	54 (36.7)	
HCV, *n* (%)	5 (11.9)	23 (15.6)	
HDV, *n* (%)	2 (4.8)	2 (1.4)	
MASLD, *n* (%)	0 (0.0)	4 (2.7)	
MASH, *n* (%)	0 (0.0)	2 (1.4)	
Alcohol, *n* (%)	10 (23.8)	43 (29.3)	
PBC, *n* (%)	1 (1.4)	2 (2.4)	
Unknown, *n* (%)	7 (16.7)	16 (10.9)	
Cirrhosis and decompensation			
Cirrhosis, *n* (%)	27 (64.2)	109 (73.6)	0.208
Hepatic decompensation, *n* (%)	7 (19.4)	40 (31.0)	0.174
Ascites, *n* (%)	7 (19.4)	35 (27.1)	0.349
Variceal bleeding, *n* (%)	2 (5.6)	13 (10.2)	0.398
Tumor Characteristics			
Single tumor, *n* (%)	25 (69.4)	61 (48.8)	0.029
PVT, *n* (%)	5 (12.8)	18 (13.8)	0.870
Extrahepatic metastases, *n* (%)	2 (5.7)	6 (4.8)	0.834
Within Milan criteria, *n* (%)	18 (42.9)	53 (36.1)	0.641
Treatment Modalities			
Hepatectomy, *n* (%)	12 (36.4)	23 (25.3)	0.225
TACE, *n* (%)	21 (52.5)	53 (40.2)	0.167
RFA, *n* (%)	13 (32.5)	45 (35.7)	0.710
Sorafenib, *n* (%)	12 (30.0)	35 (26.9)	0.704
Atezolizumab/Bevacizumab, *n* (%)	3 (9.4)	10 (13.3)	0.566
Lenvatinib, *n* (%)	2 (5.3)	5 (4.3)	0.815
Regorafenib, *n* (%)	4 (10.3)	8 (6.8)	0.488
Cabozantinib, *n* (%)	4 (10.8)	7 (6.0)	0.320
Nivolumab, *n* (%)	1 (2.7)	5 (4.3)	0.655
Child–Pugh score, mean ± SD	5.9 ± 1.2	6.1 ± 1.6	0.526
MELD-Na score, mean ± SD	10.3 ± 3.4	11.4 ± 4.5	0.330
AFP at baseline (ng/mL), median (IQR)	264 (89–7950)	275 (67–1668)	0.450

Abbreviations: BMI, Body mass index; IQR, Interquartile range; HBV, Hepatitis B virus; HCV, Hepatitis C virus; HDV, Hepatitis D virus; MASLD, Metabolic dysfunction-associated steatotic liver disease; MASH, Metabolic Dysfunction-Associated Steatohepatitis; PBC, Primary biliary cholangitis; PVT, Portal vein thrombosis; TACE, Transarterial chemoembolization; RFA, Radiofrequency ablation; SD, Standard deviation; MELD-Na, Model for End-stage Liver Disease Sodium; AFP, alpha-fetoprotein.

**Table 3:** Univariate Cox regression analysis of predictors of overall survival.

	Univariate Analysis (*p*-Value)	HR (95% CI)
Gender	0.146	1.50 (0.87–2.57)
Diabetes mellitus	0.448	1.16 (0.79–1.70)
Arterial Hypertension	0.553	0.90 (0.63–1.28)
Dyslipidemia	0.206	0.74 (0.46–1.18)
Alcohol use	0.841	0.99 (0.87–1.12)
Smoking	0.497	0.96 (0.84–1.09)
BMI	0.389	1.0 (1.0–1.0)
Cirrhosis at baseline	0.693	1.08 (0.73–1.61)
Decompensation at baseline	<0.001	3.06 (1.98–4.72)
Variceal bleeding at baseline	0.003	2.68 (1.41–5.09)
Ascites at baseline	<0.001	2.92 (1.88–4.53)
Single tumor	0.002	1.15 (1.05–1.25)
Extrahepatic metastasis at baseline	<0.001	3.84 (1.82–8.11)
AFP	0.871	1.0 (1.0–1.0)
Within Milan Criteria	0.610	0.95 (0.76–1.17)
PVT	<0.001	3.18 (1.80–5.60)
Hepatectomy	<0.001	0.32 (0.20–0.54)
TACE	0.326	1.2 (0.83–1.73)
RFA	<0.001	0.44 (0.30–0.66)
Sorafenib	0.310	0.82 (0.55–1.21)
Atezolizumab/Bevacizumab	0.202	0.64 (0.32–1.28)
Lenvatinib	0.454	0.73 (0.32–1.67)
Regorafenib	0.202	0.67 (0.36–1.24)
Cabozantinib	0.155	0.59 (0.29–1.22)
Nivolumab	0.547	0.78 (0.34–1.78)
Statin Use	<0.001	0.45 (0.28–0.7)

Abbreviations: HR, hazard ratio; CI, confidence interval; BMI, body mass index; AFP, alpha-fetoprotein; PVT, portal vein thrombosis; TACE, transarterial chemoembolization; RFA, radiofrequency ablation.

**Table 4:** Multivariate Cox Regression Model including Statin Therapy, BCLC Classification, Diagnostic era, and MELD-Na Score.

Variable	HR	95% CI	*p*-Value
Statin therapy	0.436	0.194–0.980	0.045
BCLC classification at diagnosis	1.155	0.909–1.468	0.239
Diagnostic era	1.199	0.795–1.808	0.387
MELD-Na score at baseline	1.164	1.059–1.280	0.002

Abbreviations: HR, hazard ratio; 95% CI, 95% confidence interval; Barcelona-Clinic Liver Cancer (BCLC) Staging; MELD-Na, Model for End-stage Liver Disease Sodium.

**Table 5:** Expanded multivariable Cox regression model for overall survival.

Variable	HR	95% CI	*p*-Value
Statin therapy	0.355	0.186–0.677	0.002
BCLC classification at diagnosis	1.079	0.885–1.316	0.450
Child–Pugh score at baseline	1.215	1.078–1.368	0.001
PVT at baseline	3.167	1.702–5.896	<0.001
Diagnostic era	1.040	0.764–1.414	0.804

Abbreviations: HR, hazard ratio; 95% CI, 95% confidence interval; Barcelona-Clinic Liver Cancer (BCLC) Staging; PVT, Portal vein thrombosis.

## Data Availability

The data supporting the findings of this study are available from the corresponding author upon reasonable request.

## Abbreviations

The following abbreviations are used in this manuscript:

HCC	Hepatocellular carcinoma
MASLD	Metabolic dysfunction-associated steatotic liver disease
ALD	Alcohol-related liver disease
CEUS	Contrast-enhanced ultrasound
CT	Computed tomography
MRI	Magnetic resonance imaging
BMI	Body mass index
AFP	Alpha-fetoprotein
Meld-Na	Model for End-stage Liver Disease Sodium
PVT	Portal vein thrombosis
BCLC	Barcelona Clinic Liver Cancer classification
TACE	Transarterial chemoembolization
RFA	Radiofrequency ablation
OS	Overall survival
SD	Standard deviation median with
IQR	Interquartile range
HR	Hazard ratio
CIs	Confidence intervals
HBV	Hepatitis B virus
HCV	Hepatitis C virus
HDV	Hepatitis D virus
MASH	Metabolic Dysfunction-Associated Steatohepatitis
PBC	Primary biliary cholangitis
HMG-CoA	Hydroxymethylglutaryl-coenzyme A
NF-κB	Nuclear factor kappa-light-chain-enhancer of activated B cells (NF-κB) pathway
NLRP3	Pyrin domain-containing protein 3 inflammasome
